# The impact of crowd effects on home advantage of football matches during the COVID-19 pandemic—A systematic review

**DOI:** 10.1371/journal.pone.0289899

**Published:** 2023-11-16

**Authors:** Sihang Wang, Yang Qin

**Affiliations:** 1 Department of Physical Education, Foshan Polytechnic, Foshan, Guangdong, China; 2 Department of Physical Education, Tianjin University of Sport, Tianjin, China; Instituto Politecnico de Viana do Castelo, PORTUGAL

## Abstract

This review aimed to (1) analyze whether the home advantage was diminished; (2) investigate the impact of the crowds’ support on the match outcome and team performance; (3) explore the bias of the referee without crowds. Based on the PRISMA 2020 (Preferred Reporting Items for Systematic Reviews and Meta-analyses) guidelines, this systematic review searched of literature was conducted in December 2022. The keywords related to soccer, COVID-19 and home advantage were used. The search returned 135 articles. After screening the records against set criteria, 28 articles were analyzed. Results showed that the absence of crowds would reduce the home advantage of teams, but the findings varied by country. Most leagues have evidence that without the support of the spectators, their technical, tactical and physical performances would be worse. The referee seems likely to be fairer when the spectators are absent. Therefore, crowd supports is an important factor affecting home advantage, the clubs can at least try to strengthen the home advantage and increase the winning possibility by encouraging spectators to enter the stadium or reducing the ticket price.

## Introduction

Since the birth of sports, fair competition has always been the theme of sports competitions, the core of which is to ensure that the participants play under fair and just conditions, so that the athletes’ competitions are not disturbed by external condition. However, with the expansion of sports influence and the increasing frequency of sports events, there seems to be a potential relationship between the match location and outcome, especially in home and away sports competitions [1]. Koppet (1972) studied a large number of competitions and was first put forward the definition of home advantage, which refers to a statistical phenomenon related to the match location and outcome of competitions [2]. In other words, a team playing at home is more likely to win the game than the away teams.

The study of home advantage in sports competitions is of great practical significance to exploring the winning rules of competitive sports. It has been suggested that home advantage exists in basketball, baseball, hockey, rugby and football, but football has the highest home advantage, which is nearly 10% higher than the professional basketball leagues, and nearly 15% higher than the professional baseball leagues [3]. Pollard (2005) analyzed the factors influencing home field advantage in football matches and concluded that the factors affecting home field advantage are related to the quality of the team itself, in addition to the number of spectators, familiarity with the field, player psychology, and referee bias [4, 5].

In the study of crowd effects in home advantage, Goumas investigated the goal scored in four continental confederations of the International Federation of Association Football (FIFA). It was found that the home advantage increased by 1.5% for each 10% increase in spectators size [6]. A study on the Australian A-League supports the findings that the home advantage of teams increases with increasing spectator sizes [7]. However, a key limitation of the above studies has often been noted by researchers [8, 9], is that they rarely can investigate home games without the presence of spectators. This is a serious problem, as crowd size is often considered to be a major contributor to home advantage. There has never before been an opportunity to study the impact of silent conditions on team performance within multiple leagues/countries at the same time. There existed the only known study to investigate the crowds effect on the HA was in a Italian league matches where spectators were not permitted due to security reasons [10].

The COVID-19 pandemic outbreak has postponed or even cancelled many sporting events for public health reasons, with football, the game with the largest live spectators, being the most severely impacted [11]. The 2019–2020 season, which has already started in many countries, was interrupted for about three months and then resumed, while leagues that have not yet started were postponed until the epidemic situation in each country improved and then restarted. In addition, the severity of the virus varies from country to country, as does the policy of epidemic prevention [12]. The Bundesliga was the first to restart the matches, but they banned spectators from entering and canceled pre-game ceremonies such as handshakes, a move that was followed by other European countries [13]. Even some national leagues have cancelled the home and away systems, the Argentine Super League and the Chinese Super League have arranged matches in neutral venues in a specific city, and the Korean professional football league has reduced the number of round robin matches in the regular season [14, 15]. These practices provide a unique opportunity to further investigate the impact of crowd support on match outcomes. The influence of home advantage on technical and physical performance has been a widely studied topic in professional football. For example, home teams exhibit greater running demands [16], higher total distance covered [17], and greater deceleration [18] compared to away teams. In terms of technical and tactical indicators, previous studies found that home teams has more passes [19], shots [20, 21], goals [22], and performs better in the variables related to ball possessions [23–27] than away teams. However, the crowd is one variable of the home advantage, whether the absence of the audience will affect the home advantage of the teams remains to be discussed and analyzed.

Since the outbreak of COVID-19 pandemic, it has had a great impact on team sports, especially football match which has the largest number of spectators on site. Although many studies have investigated the crowd effects recently [12, 13, 28, 29], there is no systematic review on this kind of topic. Therefore, the aim of this study attempts to systematically review the crowd effects from the following aspects: (1) analyze whether the home advantage was diminished; (2) investigate the impact of the crowds’ support on the match outcome and team performance; (3) explore the referee bias without crowds.

## Method

### Design and search strategy

The systematic review of articles examining the impact of crowd effects on match outcome and match performance during the COVID-19 pandemic was conducted according to the PRISMA statement. The search was completed on 18th December 2022 and there is no restriction on the publication date of the retrieved articles. The databases of Web of Science, Pub Med, and SPORTDiscus were searched by using the words “football or soccer”, “COVID-19” combined with each of the following keywords (‘home advantage’, ‘crowd or spectator’, ‘match performance’, ‘physical performance’, ‘technical performance’ and ‘tactical performance’). Accordingly, the results of all the databases were combined to generate the overall search outcomes. Finally, to ensure maximum retrieval of articles, the keywords in all fields were searched, and extracted the needed information for this study.

### Inclusion and exclusion criteria

The inclusion and exclusion criteria were based on the PICOS method and the details were as follows: (1) it was a relevant study of home advantage during the COVID-19 pandemic; (2)the research is related to the match outcome or match performance; (3) the language of the article was English. Articles were excluded if they had the following. (1) the match sample was a non-professional football match; (2) the study was not related to home advantage; (3) the study was not supported by data; (4) it was a conference abstract; (5) the time period of the match is not during the COVID-19 pandemic.

If there was a disagreement on the inclusion of articles between the two independent reviewers, the final decision was delivered to the senior author (CZ) due to his greater experience on these matters. In the process of screening articles, the assessment of eligibility of the articles was performed by one review author (YC). All articles were screened from titles and abstracts. Once there is ambiguity or indecision, two other reviewers will be invited to judge the disagreement, and the differences between the inclusion or exclusion of research will be resolved through consensus.

### Quality of the articles

After the inclusion of all literature, the quality of the articles was assessed in terms of the following aspects, derived from previous studies [30]: (1) the purpose of the study; (2) relevant literature review; (3) rationality of research design; (4) participants; (5) rationality of sample size; (6) informed consent; (7) reliability and validity of measurement results; (8) detained description of experimental method; (9) research results; (10) analysis of research methods; (11) theoretical connection; (12) conclusion; (13) implication. The binary scores of each item were then added to calculate the final score and presented as a percentage to reflect the quality standard of the article. The scoring criteria were categorized as follows: research methods scores pass < 50%; research methods score good between 51% and 75%; and research methods scores excellent >75%. The scoring and classification methods used in this paper are consistent with the statistical methods used in previous systematic reviews [31, 32]. An independent reliability analysis of inter-rater quality scores was performed by calculating Cohen’s Kappa values [33].

### Data extraction

The data in each article were extracted by one review author and checked by another. If there were disagreement in the exclusion, classification and selection of variables. A more experienced expert in this field will be invited to judge the arguments until all authors reach an agreement. The main information was extracted from each included study: (1) the study sample, i,e., the season and location of the leagues, the number of players and matches; (2) the purpose of the study; (3) variable analyzed, including the match outcome, match location, technical and physical variables; (4) main results, the impact of crowd support on match outcome or team performance.

## Results

**Fig 1:**
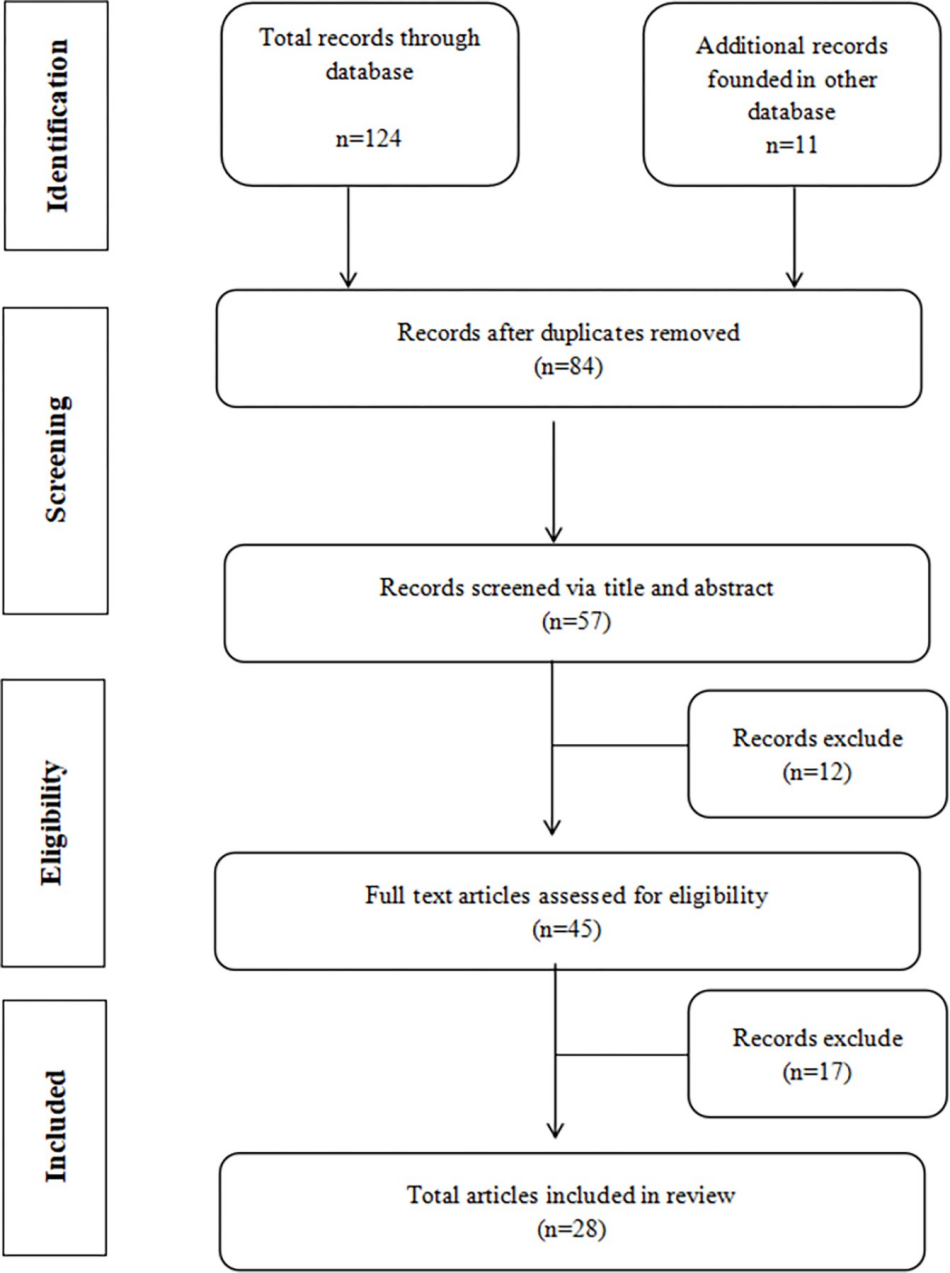


By searching keywords on the Web of Science, Pub Med, and SPORTDiscus, 135 articles were initially searched, and then 51 duplicate articles were eliminated. Then the articles were screened out based on the titles and abstracts. After excluding the studies with a small sample size and no available statistics, 17 articles were excluded. Eventually, a total of 28 articles were comprehensively reviewed. The process of screening the primary documents (see Fig 1) is shown in the following PRISMA flow diagram.

### Study quality

In previous studies, Sarmento et al. (2014) have verified its reliability and validity through the quality of literature retrieved from the Web of Science [34]. In this study, the average quality score is 90.4% after evaluating all the research methods and statistics of these studies. The quality scores of most studies were below 100%, and few were below 50%. In this paper, there are 2 articles with average quality scores (51%-75%), and 26 articles with high-quality scores (> 75%). The kappa index calculated by the reliability and validity test is 0.91, which shows that the raters have high consistency. The main defects in these studies include unreasonable sample size and improper research methods.

### Study characteristics

In all the included studies, 64.8% (18) of the articles used only the 2019–2020 season as their study sample, with this particular season being interrupted for a time due to the COVID-19 pandemic. While others analyzed the comparison between this special season and the previous seasons, which can be traced back to 2002 at the earliest [35]. The sample size ranged from 20 to 33,796 analysed matches, which consisted of 41 professional leagues from 30 different countries. Among them, 19 studies were conducted in the German Bundesliga [36–47, 50], followed by Spanish La Liga (17) [12, 14, 36–43, 45–49, 51, 52], Italian Serie A (16) [14, 35–43, 45–49, 53], English Premier League (14) [14, 36–43, 45–49], Portuguese first league(8) [39, 40, 42, 45–48, 53], etc. Besides, the UEFA Champions League (2) [35, 49]and UEFA European Football Championship (1) [35] were also analyzed. All the participants are professional male football players. In addition, regarding the impact of playing conditions without spectators on a team’s home advantage, 8 studies concluded that the impact was reflected in the match outcome [36–39, 53–55], while 6 studies was about technical and tactical performance [14, 40–44], 4 studies was about physical performance [29, 30, 50, 51], and 10 studies was related to the referee bias [13, 15, 28, 29, 45–50]. The variables of technical and tactical performance include: shots on goal, shots on target, goals scored, goals conceded, number of passes, pass accuracy, ball possessions, offside, crosses, and corners. Physical performance indicators include: total distance covered, high-speed running distance and times, sprint distance, sprints, low-speed running distance and times, medium-speed running distance and times, super-speed running distance and times, and acceleration and deceleration times. Referee sanction including: fouls, red and yellow cards, penalties.

## Discussion

Home advantage has been a great topic of research in the field of football match performance analysis, and many progress have been made [57, 58]. However, with the outbreak of COVID-19 pandemic, spectators were forbidden to enter the stadium, which provided a natural experiment to examine the crowd effect. In this condition, it may contribute to gaining a comprehensive understanding of the role of crowds in football matches.

Spectators have been described as the twelfth man in the football match, and it is obvious that spectator participation in the match also has a significant impact on the performance of players and even teams [59]. There have been many studies on crowd support [6, 7, 60], but the research conditions have not allowed for an accurate quantification of the importance of crowd support. With the outbreak of COVID-19, in order to prevent the spread of the virus, many football leagues have prohibited crowds from entering the stadium, which provides us with natural experimental conditions to investigate the influence of crowd support on team performance.

### Match outcome

The symptom of a team’s home advantage is to win more home matches. Previous studies show that, the winning possibility of home teams is over 50% [2]. Would the home matches still retain this advantage in the absence of crowds? McCarrick et al. (2021) found that the home teams won an average of 0.39 points more than away teams before the COVID-19 pandemic [9].

As shown in Table 1, many previous articles have supported this statement, that home advantage depends mainly on the support of crowds [13, 35, 37, 43, 48]. Tilp and Thaller (2020) investigated the matches in the Bundesliga in the 2019–2020 season and found that the absence of spectators significantly weakened the home advantage of the Bundesliga, the wins of away teams were higher than the home teams during the tournament [13]. McCarrick et al. (2021) compared 4,844 matches in 15 leagues in 11 countries, it was found that the home teams won fewer matches without spectators, and its home advantage decreased significantly [9]. Tugbay Inan (2020) also confirmed the significant role of the crowd’s support for home advantage by comparing the five major leagues in Europe [37]. The reason behind this may be explained by the theory of “social facilitation”. Allport claimed that the existence of other people can interfere with individual performance [61]. The away team will gain greater psychological stability when the crowds are absent, so that they will not be disturbed by the negative social pressure, thus improving the away players’ performance and weakening the home advantage [11].

**Table 1:** The impact of crowd effect on the match outcome.

Authors	Sample	Purpose	Variables	Main results	Quality score(%)
Jiméne and Lavin(2020)[36]	2442 matches from the 2019–2020 seasons from eight European elite leagues.	Investigate the impact of crowd support on the difference of wins, points and goals.	Crowd support, goals, points, stadium attendance, stadium budget, experience.	There are no significant differences between playing with or without spectator except German and Spanish leagues.	96.8
Inan (2020) [37]	8530 matches from 2015–2019 seasons in all major European football leagues.	Verify the effect of spectator’s support on home advantage.	Match outcome, defensive performance, offensive performance, running performance, spectators’ support	The spectators’ support plays a significant role in home advantage.	94.8
Ribeiro et al., (2022) [54]	2280 matches from 2018–2020 in Brazilian professional championship.	Examine the influence of spectators on the home advantage.	The number of wins, points won, goals, goal concedes, attendance.	The home advantage was diminished when the crowds absent. But the change is not obvious in lower league.	94.6
Ferraresi and Gucciardi (2020) [38]	828 closed matches from 2019–2020 season in the in European elite football leagues.	Explore the influence of crowd support on the team performance.	Match location, match outcome, total points, audience, international experience.	The performance of the home team would deteriorate without crowds. Especially those with high attendance rate and lack of experience.	94.2
Levental et al., (2022) [55]	4030 matches in the Israeli top football leagues.	The impact of crowd’s absence on the home advantage.	Match outcome, goals, goal concede, points, the crowd support, density, geographic region, league level.	The crowd support does not play a significant role in home advantage	91.2
Matos et al., (2021) [53]	34-round matches from 2019–2020 season in the Portuguese football league.	Compare the home advantage differences between the pre and post COVID-19 lockdown.	Match location, crowd support, wins, home advantage score,	The absence of spectators does not affect the team’s home advantage.	90.6
Benz and Lope (2020) [39]	17 national football leagues during the 2019–2020 seasons	Verify the change of home advantage during the COVID-19 pandemic.	Match outcome, goals, points, wins, red and yellow cards.	The changes of home advantage varied by country.	84.7
Leitner and Richlan (2020) [56]	20 matches of FC Red Bull Salzburg from 2018–2019 and 2019–2020 seasons	Compare the emotional behaviour differences in the players, officials and staff without crowds	Emotional situation of players, staff and referee including self-adaptor, protest, words fight, discussion and fair-play-behaviour	The closed match has a significant impact on the behaviour of players, staff and officials. Referees will be less motivated, players, and officials will behave more sensibly.	74.6

However, Ribeiro et al. (2022) analyzed matches in Brazilian top and second-tier professional football leagues in 2018–2020 and found that, despite the decline in home advantage in the top leagues in 2019 and 2020, surprisingly, fewer home wins took place in 2019, not in 2020 when the spectators were absent. On the other hand, in the second league, there has not been a significant downward trend in home advantage for teams over these three years [54]. This could be related to the lower attendance of fans in the lower leagues, and therefore the athletes are used to playing in a condition that is not noisy enough and without much social pressure. This finding is in line with previous studies [46, 53], which also failed to conclude that crowd support can significantly reduce the home advantage and that crowd attendance has no effect on a team’s match outcome. Wunderlich (2021) even concluded that home advantage exists in the absence of spectators and that other factors influence a team’s home advantage [40].

### Technical and tactical performance

Before the COVID-19 epidemic, many studies have fully confirmed that home advantage has a significant impact on the team’s technical and tactical performance. See Table 2, Liu et al. (2021) found that home advantage has a significant influence on ball possession and scoring first of home teams [62]. Díez et al. (2021) also reported that the team would perform better technical and tactical performance when against away teams. However, under the background of the epidemic blockade, whether the home advantage of the team is weakened needs further study [23].

**Table 2:** The impact of crowd effect on the technical and tactical performance.

Authors	Sample	Purpose	Variables	Main results	Quality score(%)
Wunderlich et al., (2021) [40]	Over 4000 matches before and after the COVID-19 lockdown in European elite leagues.	Analyse the home advantage in the absence of spectators during the COVID-19 lockdown.	Match location, crowd support, goals, points, except points, shots, shots on target, fouls, yellow cards, red cards.	The shots, shots on target, fouls, red and yellow cards reduced without crowds.	96.8
Cross and Uhrig (2020) [41]	15906 matches from 2009–2020 seasons in European football leagues.	Investigate the influence of spectator attendance on the home advantage.	Match location, match outcome, spectator attendance, goals, points, distance covered.	The absence of spectators reduce 50% goals of home teams.	95.4
Almeida and Leite (2021) [42]	982 matches from 2019–2020 season in German Bundesliga, LaLiga, English Premier League, Portuguese Primeira Liga and Italian Serie A	Investigate the impact of COVID-19 lockdown on the home advantage and team performance.	Points, goal scored, goal conceded, total shots, shots on target, ball possession, pass success, aerial duel won, tackles, cards.	The lockdown had an impact on the team performance, as evidenced by a significant decrease in shots, tackles, shots on target, and passing success.	93.8
Hill and Van Yperen (2021) [43]	5784 matches from 2015–2020 seasons in the German Bundesliga, LaLiga, English Premier League, Italian serie A.	Validate the impact of crowd support on home advantage.	Crowd support, points, goals, shot, possession, fouls, yellow cards and red cards.	Home advantage may indeed be lost when the spectators was absent. The goals of away teams increased and home teams got more yellow cards.	91.6
Chen et al., (2022) [14]	397 matches from 2019–2020 seasons in the Chinese Soccer League.	Compare the technical and physical performance differences without crowd supports	Crowd support, total distance, sprint distance, pass, possession, shots, cross, fouls, offside, team quality, opponent quality	The cross,shots and shot success was decreased without spectators.	90.4
Santana et al.,(2021) [44]	305 matches from 2019–2020 Bundesliga seasons.	Investigate the changes in the match and physical performance variables pre and post COVID-19 lockdown.	Goals, Ball possession, Passes, Passes accuracy, Distance, Sprints, Tackles won, Corners, Offside, Foul committed	The matches without crowds can diminish the home advantage and on technical performance.	84.8

During the COVID-19 epidemic, many studies have found that in several European football leagues (Bundesliga, La Liga, Premier League, Portuguese first league, Italian Serie A), the number of goals, shots, and shots on target of home teams are significantly decreasing after the COVID-19 lockout [9, 12, 40–42, 44, 46]. This finding is in line with Chen et al. (2022) [14], which found a significant decrease in pass, shot, and shot success rates in the Chinese Super League. The reason for the decline of the team’s technical performance may lie in social factors. When the spectators are present, enthusiastic cheers and slogans can motivate players, stimulate their territorial awareness and offensive aggression, and make their performance better [42]. However, with the absence of the audience and the loss of spectators’ attention and encouragement, the crowd effect also dissipated.

While Gomez (2016) holds a different opinion, that is, the crowd support has little influence on The technical and tactical performance of players, because the players’ aggressiveness, territoriality and familiarity with the stadium are the crucial factors that cause the home advantage [63]. In the top leagues, there are many players who come from other countries, and they are used to playing in unfamiliar countries and cities, so the crowd effect will not significantly affect their match performance [64]. However, in the lower levels of the league, most players are domestic players, and even growing up from the local youth training system, they have a stronger sense of territory when playing at home, which leads to their more aggressive and better performance [36].

### Physical performance

The most significant impact of the COVID-19 pandemic on football players is undoubtedly the physical aspect. With the infection and closed-door training conditions, the physical activity ability, cardiopulmonary and respiratory function of players were affected. Which would have a significant impact on the players’ physical performance in the game.

As Table 3 depicted, Raya-González et al. (2022) compared the running performance of La Liga before and after the COVID-19 pandemic. The results showed that the total distance, the distance at all intensities, and the times of acceleration and deceleration have all decreased. Especially, the greater the decline in running performance, the lower the ranking of the team [29]. Santana et al. (2021) explored the running performance of the Bundesliga before and after the lockout, it was found that the home team sprinted more distance than the away team, but the total running distance decreased [44]. This finding is consistent with previous research results [52, 65]. The reason may be that after long-term individual training, the time for team training is reduced, and players lack friendly matches to maintain good physical condition. Besides, the more congested schedule also aggravates the fatigue of players, leading to the decline of players’ running ability. On the other hand, with the large-scale spread of the COVID-19 epidemic, many players failed to escape the infection, which had a negative impact on their cardiopulmonary health and running performance [66, 67].

**Table 3:** The impact of crowd effect on the physical performance.

Authors	Sample	Purpose	Variables	Main results	Quality score(%)
Raya-González et al., (2022) [29]	23257 individual match observations from 2019–2020 seasons in the LaLiga.	Investigate the running performance difference between the pre and post COVID-19 lockdown.	Total distance covered, distance covered at 21–24 kmh, high metabolic load distance, accelerations, decelerations.	The running performance was decreased after the lockdown. Especially for teams whose ranking worse.	96.8
Díez et al., (2021) [23]	401 player played over 2018–2019 season and 2019–2020 season in LaLiga.	Exploring the effects of epidemic home training on players’ physical performance.	Total distance cover, low intensity distance, medium intensity, high intensity, ultra high intensity, sprint distance, substitutions, playtime.	The high intensity running distance decreased, but the total distance did not change significantly.	92.6
Rampinini et al., (2021) [12]	265 professional players from the Italian Series A in 2019–2020 seasons.	Analysed the imact of the COVID-19 lockdown on players’ physical performance.	Total distance, high-intensity distance covered, very high-speed, sprint, high-acceleration, high-deceleration.	There is no significant difference in high-intensity running performance pre and post-lockdown. But the total distance covered and very high speed decreased.	86.9
García-Aliaga et al., (2021) [52]	22 matches of pre and post lockdown of LaLiga 2019–2020 seasons.	Explore the running performance difference of pre and post-lockdown COVID-19 in LaLiga.	Duration, distance, low, medium and high speed running, high-intensity actions, accelerations, decelerations, sprint speed running.	Running performance was superior in the pre-lockdown phase, including medium, high and sprint speed running. However, the number of accelerations and decelerations increased significantly post-lockdown period.	79.2

However, there are also some studies with opposite opinions. Raya-González et al. (2022) found the number of sprints and total distances of players increased significantly in the matches without spectators [29]. García-Aliaga et al. (2021) pointed out that there is no significant decrease in the total distance and sprint distance, but the number of accelerations and decelerations increased significantly during the post-lockdown period. Possible explanations for this are that the players’ physical and technical conditions have declined because of home-based training, which would increase the possibility of making mistakes and give opponents more opportunities to counterattack quickly [52]. A study on the Chinese Super League (CSL) has reached a similar result that the total and sprint distance of teams is increasing when the spectators were absent [14]. The reason may be that the CSL divided all teams into two tournament areas to reduce the possibility of infection, and all teams play in a specific place. This measure eliminates the effects of home advantages, travel fatigue, and congested schedules, which may lead to abundant energy in physical performances to prepare for the upcoming games.

### Referee bias

Referee bias is considered to be the key factor to determine the result of the game, and they do their best to ensure the fairness of the game. However, many studies showed that crowds would influence the referee’s decision (see Table 4). McCarrick et al. (2021) compared the referee behavior pre and post-COVID-19 lockdown from the 2019–2020 seasons in the 15 different football leagues. It was found that the absence of spectators had a significant impact on the bias of referees. The fouls and yellow cards of away teams are reduced, but the number of red cards was not affected [45]. After analyzing the 1468 games, Bryson et al. (2020) also concluded that the referee’s bias towards the away team was more lenient in the absence of spectators, which made the away teams get fewer fouls and yellow cards [46]. Krumer et al. (2022) added that the referees’ prejudice is also affected by the audience attendance rate. The larger the audience, the more favorable the referee’s decision will be for the home team [15]. There are also some studies with opposite opinions [49, 50]. Kai Fischer (2021) investigated three consecutive season matches in the Bundesliga and found that there is no evidence that the absence of crowds makes the referee’s decisions fairer. The reason may be caused by the small sample size and league differences [28].

**Table 4:** The impact of crowd effect on the referee bias.

Authors	Sample	Purpose	Variables	Main results	Quality score(%)
McCarrick, et al.,(2021) [45]	4844 matches from 2019–2020 seasons in the 15 different football leagues.	Compare the team performance and referee behaviour pre and post COVID-19 lockdown.	Match outcome, match location, points, goals, shots on target, dominance, corners, fouls, yellow and red cards.	Without the crowds support, the home advantage was reduced, and the referee bias was diluted.	96.4
Bryson et al., (2020) [46]	6,481 football matches played before and after the shutdown in 17 countries, including 1,498 matches without crowds.	Investigate the impact of absentee crowds on refereeing decisions during the COVID-19	Home win share, Goal difference, Total goals, Home yellow cards, Away yellow cards, Yellow difference.	The number of yellow cards was significantly affected. The away team received fewer cards without crowds, which reducing the home advantage.	96.2
Cueva (2020) [47]	41 professional football leagues from 30 countries during the 1993–2020 seasons.	Examine the impact of the COVID-19 lockout on home advantage.	Match location, match outcome, fouls, goals, red and yellow cards.	The crowds have a significant impact on the referee decisions and match outcome. the home advantage reduced 50% and referee bias disappeared.	94.6
Fischer and Haucap (2021) [28]	2976 matches from 2017–2020 seasons in the three German men’s top divisions.	Explore the relation between crowd support and home advantage	Match location, crowd support, ability covariate, geographical factor, specific matches, home stadium.	There is no evidence that the absence of crowds makes the referee’s decisions more fair	94.2
Krumer et al., (2022) [15]	2160 matches from 2011 to 2019 season in Chinese Super Leagues.	Investigate the impact of crowd support on the home advantage.	Points, goals, attendance, opponent quality, match location, match outcome, red and yellow cards.	The attendance of spectators play a significant role in a home advantage, as evidenced by the goal, points and yellow cards.	92.4
Scoppa (2021) [48]	917 matches from the 2019–2020 season in Germany, Spain, England, Italy and Portugal.	Analyse the impact of crowd support and referee decision on the home advantage and team performance.	Match location, match outcome, spectator attendance, points, goals, goal difference,	The home advantage was significantly effect by the crowd support. The home advantage decreased and the referee decision tend to more fair in closed game.	92.2
Sors et al., (2021) [49]	841 close matches from 2019–2020 seasons in UEFA, Spain, England, Germany and Italy football leagues.	Investigate whether home advantage and referee bias still exist in matches without spectators	Match outcome, points, goals, ball possession, shot, corners, fouls, yellow and red cards, penalty.	The crowd support plays a important role in home advantage and referee bias.	90.8
Reade et al., (2021) [35]	33796 matches from 2002–2020 seasons in European elite matches.	Examine the impact of social pressure from spectators on referee behavior and match outcome.	Attendance, home elo rating, away elo rating, home win, draw, away win, yellow cards, red cards, penalty kicks.	There is no significant effects on match outcome. But the home advantage is reduced, and the punishment of away teams was significantly decreased by referee.	88.4
Tilp and Thaller (2020) [13]	306 football matches in the 2019–2020 Bundesliga season, 223 were played with spectators and 83 without spectators.	Analyse the impact of empty games on player performance and refereeing behaviour.	Match outcome, fouls, red cards, yellow cards and penalty.	The home advantage was significantly reduced in the absence of the crowd.	82.6
Dilger and Vischer (2020) [50]	83 matches from 2019–2020 seasons in the German football league.	Verify the impact of crowds on home advantage	Match location, match outcome, points, goals, shots on target, distance, passes, yellow and red cards.	The referee bias disappeared and they gave significantly fewer yellow and red cards to the away team.	73.8

Besides, it seems that there are technological reasons for the reduction of referee bias. Many European football leagues have started to use Video Assistant Referees (VAR). It provides visualization from different angles, which is conducive to reducing some decision-making mistakes and reducing the impact of social pressure caused by the crowd. The application of VAR has changed the dynamics of football leagues [68], and even lead to a decrease in HA in some cases [69]. In addition, there are many reasons for the referee’s bias, such as experience [70], height [45], and physical condition [55]. Although the referee is an official who maintains the fairness of the game, in most cases, their decision is more inclined to attack.

The limitation of the present study mainly lies in the small number of matches without crowds in each league, and the containing measures varied from leagues during the COVID-19 pandemic. The league differences can make it difficult to analyze the sample size across leagues. In addition, the COVID-19 epidemic also affects the match performance, including infection [66, 67], home-based training [52], rules change [39] (the number of substitutions increasing from 3 to 5) and congested schedule [53] may lead to the decline of the team’s home advantage. Therefore, future research needs to address these issues.

## Conclusion

The purpose of this systematic review was to reveal the impact of the crowd effect on the team’s home advantage during the COVID-19 pandemic. In the match outcome, according to the theory of social psychology, many studies found that the absence of spectators will reduce the winning rate of the home teams, and home advantage has been weakened or even disappeared, and this trend is particularly obvious in the Bundesliga. In terms of technical and tactical performance, with the decline of the score, the offensive indicators of home teams are even worse, such as the fewer number of goals, shots, shots on target and ball possessions, while the away teams performed better. Because of the effect of home-based training and virus infections, many studies have pointed out that the total distance, high intensity running distance of most teams has decreased, but the difference in speed is not obvious. No evidence shows that physical performance of players is related to crowd support. With regard to referee bias, many studies have found that the referee is more tolerant of the sanction of the away teams without the pressure of the spectators. However, it is difficult to confirm that the decrease in home advantage is only due to crowd effects, and it may also be related to home-based training, COVID-19 infections, rule changes, congested schedule and different epidemic prevention measures. Therefore, future research could explore the impact of these potential variables on match performance. Moreover, it is necessary to minimize the influence of non-spectator factors on the team performance by investigating a larger sample size, so as to understand the role of the crowd effect in home advantage more accurately.

## Supporting information

S1 Checklist PRISMA 2020 checklist. (ZIP)
